# The complete chloroplast genome sequence of *Prunus sibirica*

**DOI:** 10.1080/23802359.2019.1710589

**Published:** 2020-01-14

**Authors:** Shengjun Dong, Xin Zhang, Yongqiang Sun, Hao Xu, Haokai Zhang, Jianhua Chen, Mingguo Liu

**Affiliations:** a School of forestry, Shenyang Agricultural University, Shenyang City, Liaoning, China;; b State Key Laboratory of Tree Genetics and Breeding, Northeast Forestry University, Harbin, China

**Keywords:** *Prunus sibirica*, chloroplast genome, phylogenetic tree

## Abstract

In this study, the chloroplast genome sequence of *Prunus sibirica* was obtained from the whole genome sequencing data of *Prunus sibirica*. Its length is 158,248 bp, which consists of 86,331 bp large single-copy region (LSC), 26,408 bp two reverse repeat regions (IR) and 19,101 bp small single-copy region (SSC). GC content of the whole chloroplast genome is 36.71%. Those of LSC region, SSC region, and IR region were 35, 30, and 43%, respectively. There are 131 unique genes in the chloroplast genome, including 90 protein-coding genes, 33 tRNA genes, and 8 rRNA genes. A maximum-likelihood phylogenetic tree was generated from the chloroplast genomes of 10 species of *Rosaceae* and 11 peripheral plants. The results showed that *Prunus sibirica* belongs to *Rosaceae* and is sister to *Prunus salicina*.

Siberian apricot is a joint name of wild apricot. Nine apricot species have been identified in China, among which *Prunus sibirica*, *P. mandshurica,* and *P. vulgaris* are the most generous. Siberian apricots are significant ecological and economic trees, which are light loving, cold resistant and barren resistant. It is the preferred trees for afforestation in arid and semi-arid areas. Its economic product is mainly bitter apricot and found to be in short supply. However, the low and unstable output has become the main bottleneck restricting the industry, which needs thorough research at a population level to provide high and stable yield varieties for the industry (Li et al. 2013, 2018).

The *Prunus sibirica* leaves samples were collected in Zhalantun (Inner Mongolia, China; 47°54′N, 120°48′E) and stored in the Herbarium of Shenyang Agricultural University as a voucher specimen (AS2019061-1), and fresh leaves total DNA was isolated by modified CTAB method (Doyle and Doyle 1987) in the herbarium. The Illumina library was constructed and sequenced on Hiseq 2000 (Illumina, San Diego, CA, USA).

For generated 199,953,290 reads, the genome was assembled by Velvet (Zerbino and Birney 2008), NOVOPlasty (Dierckxsens et al. 2016), and SSPACE (Boetzer et al. 2011). GapFiller (1000 Genomes Project Consortium 2012) was used to complement gaps in the scaffold sequence. The chloroplast was annotated by GeSeq, tRNAscan-SE (Lowe and Chan 2016), and ARAGORN (Laslett and Canback 2004). After the chloroplast genome assembly was qualified, it was submitted to GenBank (ID: MN708049).

The length of *Prunus sibirica* chloroplast genome is 158,248 bp, consists of 86,331 bp large single copy (LSC), 26,408 bp two reverse repeats (IR) and 19,101 bp small single copy (SSC). GC content of them were 35, 43, and 30%, respectively. The coding sequences (CDS) accounted for 50.36%. There are 131 unique genes, including 90 protein, 33 tRNA and 8 rRNA. Ten genes contain introns, *ycf3* and *clpP* were found with two introns. The 5′ end exon, 3′ end exon and intron of *rps12* are located in the LSC and IR region, respectively. A total of 19 genes were found in IR region, including 9 tRNAs (t*mH-GUG*, *trnM-CAU*, *trnL-CAA*, *trnV-GAC*, *trnI-GAU*, *trnA-UGC*, *trnL-CAA*, *trnN-GUU,* and *trnR-ACG*), 8 protein genes (*rps7*, *rpl2*, *rpl23*, *rps12*, *rps19*, *ndhB*, *ycf1,* and *ycf2*) and 2 rRNAs (16S and 23S RNA). At the same time, pseudogenes were formed in the part of *ycf1* and *rps19* in the IR.

A phylogenetic tree (with 1000 replicates) was constructed in MEGA 7.0, which includes 10 *Rosaceae* plants and 11 exophytes. The results showed *Prunus sibirica* belongs to *Rosaceae* (Figure 1). The relationship between *Prunus sibirica* and *Prunus salicina* is closer. Previous studies suggested that the relationship between *Prunus vulgaris* and *Prunus mume* is close (Zhang et al. 2018), but we found that *Prunus sibirica* and *Prunus salicina* is closer, suggesting there is a wide range of genetic variation in apricot plants.

**Figure 1.**
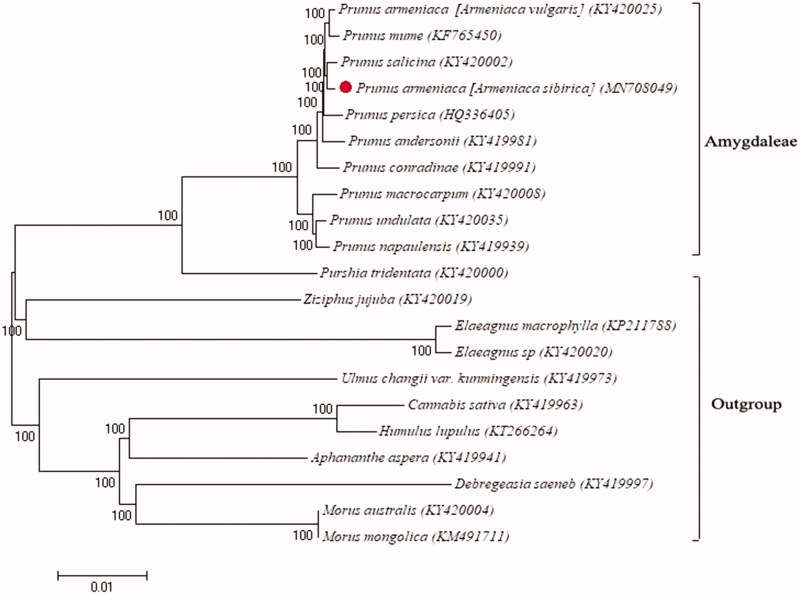
The phylogenetic tree (1000 replicates) of 10 *Rosaceae* plants and 11 Exophytes.

In conclusion, the complete chloroplast genome in this study supports the phylogeny study of *Rosaceae*.
